# Environmental Cyanide Pollution from Artisanal Gold Mining in Burkina Faso: Human Exposure Risk Analysis Based on a Conceptual Site Model

**DOI:** 10.3390/ijerph22071125

**Published:** 2025-07-16

**Authors:** Edmond N’Bagassi Kohio, Seyram Kossi Sossou, Hela Karoui, Hamma Yacouba

**Affiliations:** Laboratoire Eaux, Hydro-Systèmes et Agriculture (LEHSA), Institut International d’Ingénierie de l’Eau et de l’Environnement (2iE), Ouagadougou 01 BP 594, Burkina Faso; seyram.sossou@2ie-edu.org (S.K.S.); hela.karoui@2ie-edu.org (H.K.); hamma.yacouba@2ie-edu.org (H.Y.)

**Keywords:** artisanal gold mining, cyanide, environmental pollution, spatial analysis, site conceptual model, human exposure, health risks, Burkina Faso

## Abstract

Artisanal and small-scale gold mining (ASGM) in Burkina Faso increasingly relies on cyanide, intensifying concerns about environmental contamination and human exposure. This study assessed free cyanide levels in water and soil across three ASGM sites—Zougnazagmiline, Guido, and Galgouli. Water samples (surface and groundwater) and topsoil (0–20 cm) were analyzed using the pyridine–pyrazolone method. Data were statistically and spatially processed using SPSS version 29.0 and the Google Earth Engine in conjunction with QGIS version 3.34, respectively. A site conceptual model (SCM) was also developed, based on the literature review, field observations, and validation by multidisciplinary experts in public health, toxicology, ecotoxicology, environmental engineering, and the mining sector, through a semi-structured survey. The results showed that 9.26% of the water samples exceeded the WHO guideline (0.07 mg/L), with peaks of 1.084 mg/L in Guido and 2.42 mg/L in Galgouli. At Zougnazagmiline, the water type differences were significant (F = 64.13; *p* < 0.001), unlike the other sites. In the soil, 29.36% of the samples exceeded 0.5 mg/kg, with concentrations reaching 9.79 mg/kg in Galgouli. A spatial analysis revealed pollution concentrated near the mining areas but spreading to residential and agricultural zones. The validated SCM integrates pollution sources, transport mechanisms, exposure routes, and vulnerable populations, offering a structured tool for environmental monitoring and health risk assessment in cyanide-impacted mining regions.

## 1. Introduction

The expansion of artisanal and small-scale gold mining (ASGM) in Burkina Faso over the past decade has been accompanied by a rapid shift in extraction practices, particularly through the widespread adoption of cyanidation techniques [1]. While the use of sodium cyanide (NaCN) significantly improves gold recovery efficiency, its uncontrolled and unregulated deployment on the artisanal mining sites has introduced considerable environmental and public health risks [2,3]. Today, cyanide is used on almost all ASGM sites across the country, despite the absence of adequate containment systems and environmental safeguards [4].

Preliminary field observations and reports of aquatic fauna mortality in gold-mining regions suggest the presence of environmental cyanide contamination [5]. However, the scientific data on cyanide concentrations in the affected soils and waters remain scarce, making it difficult to establish causal links between the contamination and the observed ecological effects [6]. Furthermore, there is little information on the potential spread of pollution beyond the mining areas into agricultural lands and human settlements—areas that could represent major exposure pathways for nearby populations [7]. This knowledge gap hinders a risk assessment and the formulation of sustainable management strategies.

At the same time, concerns are mounting regarding the human exposure risks posed by cyanide. Although the health impacts of mercury in ASGM contexts have received more attention, cyanide is also a highly toxic substance, with lethal effects even at low doses. It can affect humans through multiple pathways, including inhalation of hydrogen cyanide gas (HCN), ingestion of contaminated water or soil, dermal absorption, and consumption of contaminated crops or aquatic species [8,9,10]. In the artisanal sites, where safety protocols are lacking, miners and surrounding populations [11]—including children—are frequently exposed to cyanide residue during ore processing or through proximity to polluted environments [12,13,14,15].

Amid the progressive phase-out of mercury following the Minamata Convention [16,17], the increasing reliance on cyanide underscores the urgent need to understand its environmental dynamics and associated exposure risks [18,19]. Unlike mercury, cyanide is often perceived as less dangerous due to its rapid environmental degradation. However, this perception tends to downplay the real risks posed by improper handling and chronic exposure.

This study aims to contribute to the understanding of cyanide-related environmental and human exposure risks in ASGM contexts in Burkina Faso. First, it quantitatively assesses free cyanide contamination in surface water, groundwater, and soils across three representative mining sites—Zougnazagmiline, Guido, and Galgouli. Second, it maps the spatial distribution of cyanide in relation to land use patterns. Finally, to integrate and contextualize these findings, a site conceptual model (SCM) was developed based on the literature review, field data, and expert validation. Rather than estimating dose-response health effects, the SCM focuses on identifying pollution sources, transport mechanisms, exposure pathways, and vulnerable populations. This approach provides a structured basis for future risk assessment, environmental monitoring, and the design of preventive strategies tailored to the ASGM settings in West Africa.

## 2. Materials and Methods

### 2.1. Location of Study Sites

**Figure 1:**
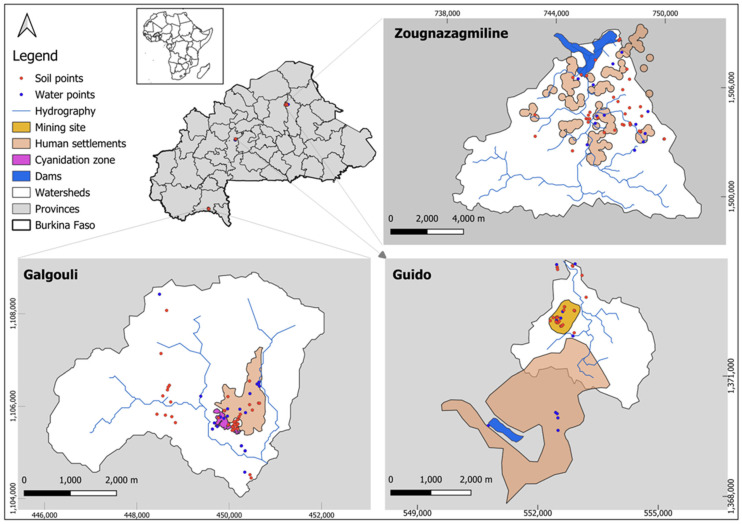
Location of the study sites and sampling points.

This study was conducted on three artisanal and small-scale mining (ASM) sites in Burkina Faso: Guido, Galgouli, and Zougnazagmiline. These sites are located in the country’s three main climatic zones, enabling a comparative analysis of pollution dynamics across distinct environmental contexts. Zougnazagmiline, situated in the commune of Bouroum (Central–North region), lies within the Sahelian zone, approximately 250 km north of Ouagadougou. Guido is located in the commune of Réo (Central–West region), representative of the Sudanian–Sahelian zone. Lastly, Galgouli is found in the commune of Kampti (Southwest region), within the Sudanian zone, about 450 km southwest of the capital, near the border with Côte d’Ivoire (Figure 1).

Data collection campaigns were carried out in the catchment areas of these sites at different periods. The data from Zougnazagmiline and Galgouli were collected between 2014 and 2016 as part of Razanamahandry’s doctoral research [20]. By contrast, the data for Guido were collected in 2021 as part of the present study, providing an updated assessment of cyanide contamination in a more recent mining context.

### 2.2. Physical Characteristics of the Study Site and History of ASGM Activities

#### 2.2.1. Zougnazagmiline Site

The Zougnazagmiline site, in the Sudanian–Sahelian zone, has a climate with two distinct seasons: a seven-month dry period (October to May) and a five-month rainy season (May to September), with annual rainfall of about 500–600 mm [21]. The Bouroum commune has a sparse hydrographic network, consisting of temporary streams, three seasonal ponds, one reservoir, and many wells and boreholes [20]. The soils are mainly tropical ferruginous (60%), followed by lithosols, hydromorphic soils, and sub-arid brown soils (BUNASOLS). Vegetation includes wooded savanna in lowland areas and shrub steppe over most of the site [20]. In this zone, livelihoods are primarily based on rain-fed subsistence agriculture (mainly millet and sorghum), traditional livestock rearing, and artisanal gold mining. These activities are often complemented by small-scale trade and handicrafts.

The ASGM site of Zougnazagmiline was discovered over 30 years ago after villagers from Kayara accidentally found gold nuggets. Classified as an illegal or “wild” site, it lacks an official exploitation permit [22]. Initially exploited in the 1980s, the site was abandoned due to rapidly depleted gold grades. In 2012, exploration by the Taparko Mining Company (SOMITA) confirmed the presence of gold, triggering a renewed wave of unregulated artisanal mining. The closure of the nearby Fillon 12 site in the same year led to an influx of miners to Zougnazagmiline, now informally managed by a local customary authority without state oversight [22].

#### 2.2.2. Guido Site

Guido village, in Burkina Faso’s Central–West region, lies in the North Sudanian climate zone with irregular annual rainfall between 600 and 900 mm. The landscape includes low plateaus and plains, reaching 400 m at Mount Sanguié [23]. The Réo commune has good water resources, notably the Vruoa-sa stream, several seasonal waterways, and a dam near the RN21 road [24]. The main soils are indurated leached tropical ferruginous soils (60%), followed by mottled types, lithosols, and hydromorphic soils in wet areas (BUNASOLS). Vegetation includes shrub and wooded savannas, and gallery forests [23]. In this commune, livelihoods are mainly based on rain-fed agriculture (millet, sorghum, maize), cash crops (cotton, sesame), traditional livestock rearing, artisanal activities, and seasonal migration during the lean period.

Guido’s gold site emerged in 2008 after a Mossi miner discovered gold in a field. Mining began without local approval, prompting intervention by village leaders. Ore processing was initially performed in Perkoa, but later shifted to Guido. A dedicated processing area was developed on land belonging to the village chief and a local elder. Though unofficial, the site is well organized under traditional leadership, with clear zoning: excavation, chemical processing, and residential areas are separated. A local rule allocates 20% of the ore to the host community.

#### 2.2.3. Galgouli Site

Galgouli village, in Burkina Faso’s Southwest region (Poni province), lies in the Sudanian climate zone with two distinct seasons: a dry season (November to March) and a rainy season (April to October). Annual temperatures range from 17 to 36 °C, and rainfall varies between 1000 and 1200 mm. The hydrographic network is dominated by the Mouhoun River and its tributary, the Poni, with additional seasonal streams and ponds [25,26]. The main soils are indurated leached tropical ferruginous soil (55%), followed by lithosols on rock and laterite (35%), and hydromorphic soil (10%) in low-lying areas (BUNASOLS). Vegetation includes wooded savannas, dense Guinean savannas, and gallery forests [26]. In Kampti, livelihoods are primarily based on rainfed agriculture focused on food crops (sorghum, maize, yam) and cash crops, such as groundnut and sesame. This is commonly combined with traditional livestock farming and widespread artisanal gold mining across the area.

Gold mining in Galgouli, initially a women-led domestic activity [27], intensified from 2005 with the arrival of migrant miners on sites, such as Bantara and Galgouli, sparking tensions with the local Loron population. These conflicts were driven by concerns over land appropriation and the desecration of sacred spaces [28]. While local authorities initially rejected the outsiders, some later accepted their presence, reportedly following symbolic gestures. In the nearby village of Dindou, migrant miners helped fund school infrastructure through voluntary contributions. The project included classrooms, a storehouse, and administrative offices. Today, a coordinator manages the site and mediates conflicts. Unlike other informal sites, Galgouli operates with an official permit and a legal gold trading center [21].

### 2.3. Water and Soil Sampling and Collection

To quantify free cyanide concentrations, systematic sampling campaigns were conducted across the study sites. Water samples were collected from various sources, including surface water (stagnant rainwater, rivers, and dams) and groundwater (wells and boreholes). Sampling was performed using a sampling rod, and the geographic coordinates of each point were recorded using a GARMIN MAP 78 GPS. Water samples were collected in 500 mL borosilicate glass bottles sterilized at 160 °C for 20 min, immediately labeled, and preserved by adding NaOH pellets to prevent hydrogen cyanide (HCN) volatilization, following APHA method SM-4500-CN-F [29]. Samples were stored in coolers with ice packs until laboratory transfer.

**Figure 2:**
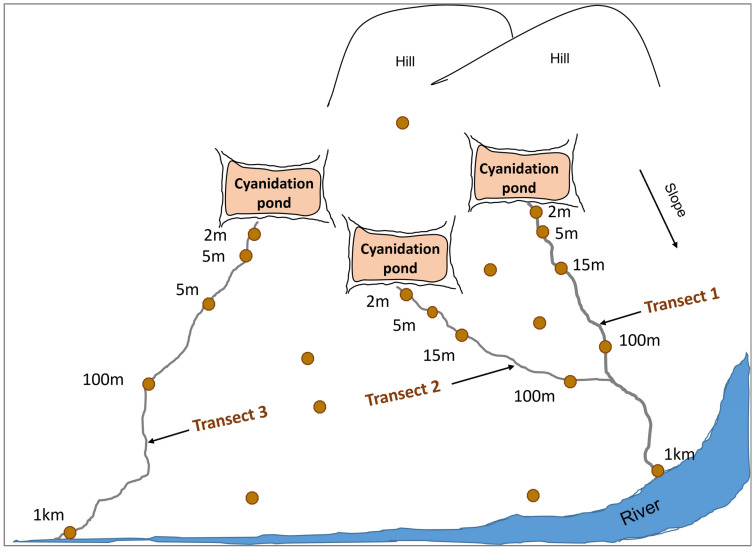
Schematic representation of the soil sampling method.

Soil sampling combined random and systematic approaches within the watershed of each site. Transects were established along erosion channels, starting from cyanidation ponds identified as primary contamination sources. Samples were collected at approximately 2 m, 5 m, 15 m, 100 m, and 1 km from these ponds. Additional random samples were taken to better capture spatial heterogeneity. Sampling site selection also considered proximity to hydraulic structures (Figure 2). Using a hand auger, samples were taken from the 0–20 cm surface horizon. Approximately 500 g of soil was collected per point and stored in labeled black plastic bags to prevent cyanide photodegradation [30]. Samples were preserved in coolers with ice to maintain their physico-chemical integrity prior to laboratory analysis.

### 2.4. Free Cyanide Quantification in Water and Soil Samples

Water samples were filtered through 0.45 µm membrane filters to clarify the solutions and to enhance UV transmittance during spectrophotometric analysis, as unfiltered samples may overestimate cyanide concentrations. A few drops of each sample were tested using potassium iodide-starch (KI-starch) paper to detect residual chlorine, a potential oxidant of cyanide [29]. Free cyanide in soil samples was extracted with 0.5 N NaOH following the method of Rennert and Mansfeldt [31], based on SMEWW Standard 4500-CN-C (1999). One gram of dry soil was mixed with 10 mL of 1 N NaOH, centrifuged at 1000 rpm for 10 min, and the supernatant was collected for analysis.

Free cyanide in both water and soil supernatants was quantified using a HACH DR 5000 spectrophotometer at 612 nm, following the pyridine–pyrazolone method (Hach Method 8027) [32]. This colorimetric method, which produces a visible color change from pink to blue proportional to cyanide concentration, has a validated detection range of 0.002 to 0.240 mg/L. Analytical accuracy was ensured through the use of certified reference materials (CRM), with recovery rates consistently falling within acceptable limits. Method detection limits were confirmed through repeated measurements of reagent blanks and low-concentration spiked samples, with a lower limit of quantification established at 2 µg/L. All analyses were performed in duplicate to assess precision and reproducibility. When cyanide concentrations exceeded the upper detection limit, appropriate sample dilutions were conducted prior to reanalysis.

### 2.5. Data Analysis

Descriptive statistics (mean, standard deviation, and range) were computed to summarize free cyanide concentrations in water and soil samples across the study sites. One-way analysis of variance (ANOVA) was performed using International Business Machines (IBM) Statistical Package for the Social Sciences (SPSS) version 29.0 to assess significant differences in free cyanide levels between water types and among sampling sites, with a significance threshold set at *p* < 0.05. All graphs were generated using R Statistical Computing Software version 4.4.1. The study site map was produced using Quantum Geographic Information System (QGIS) version 3.34.

### 2.6. Spatial Analysis Methodology for Cyanide Pollution

This study combined land use classification with free cyanide concentration mapping, integrating surface unit data. Sentinel-2 Level 2A images (November 2014–November 2019) with less than 10% cloud cover were selected for their suitability in land cover discrimination. A median composite was applied to reduce atmospheric noise. Four spectral bands (B2, B3, B4, B8) with 10 m resolution were used [33,34]. Training data were obtained through manual digitization and quality control, covering agriculture, built-up areas, natural land, and water bodies. Land use classification using the Google Earth Engine (GEE) was conducted using the random forest algorithm (500 trees, 70% sampling, 80/20 training-validation split, two variables per node), due to its strong performance on Sentinel-2 data. The accuracy assessment relied on a confusion matrix, kappa coefficient, and user’s, producer’s, and overall accuracy metrics [35]. The final land cover map was overlaid on a false-color composite and exported as GeoTIFF, consistent with environmental monitoring practices [34].

**Figure 3:**
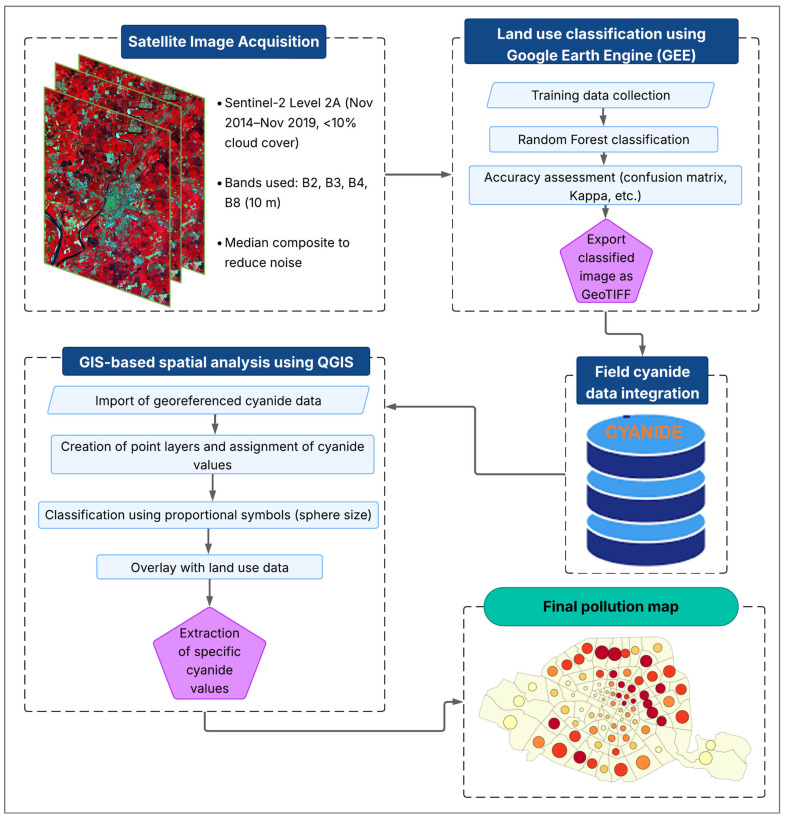
Illustration of the methodology for mapping the spatial distribution of free cyanide concentrations overlaid with land use classes for the artisanal gold mining sites in Burkina Faso.

Free cyanide concentration data, stored in a structured Excel file with geolocation, were verified, cleaned, and converted into point layers in QGIS version 3.34 (UTM WGS 84, zone 30 N). Each point was assigned a free cyanide concentration value and symbolized using proportional symbols—spherical markers whose size reflects pollution levels [36]. These symbols were classified into three pollution categories, with dark red spheres indicating highly polluted zones, orange for moderately polluted zones, and green for low pollution levels. The resulting point layer was overlaid with the land use classification to analyze spatial relationships. Specific free cyanide values were extracted by surface unit types (e.g., settlements, water bodies, agricultural land) using zonal statistics. Contaminated zones were identified based on environmental and health thresholds, supporting targeted risk characterization. This workflow aligns with standard spatial analysis procedures in environmental monitoring [34,35]. The key steps of the spatial analysis workflow are summarized in Figure 3, illustrating the integration of land use classification and free cyanide concentration mapping.

### 2.7. Development of the Site Conceptual Model (SCM)

The SCM serves as a critical framework for health risk assessment by integrating environmental and site-specific data to analyze contaminant-related hazards. It bridges field investigations and risk evaluation, thereby facilitating communication among stakeholders and guiding decision-making [37]. By identifying pollution sources, contaminant migration pathways, and potential receptors, the model provides a comprehensive understanding of site dynamics, enabling more accurate assessments and more effective risk management [38].

#### 2.7.1. Context and Problem Definition

The development of the site conceptual model for assessing human exposure risks related to cyanide use in the artisanal gold processing in Burkina Faso was based on the methodology proposed by Lanno and Menzie [39] for ecological risk assessment. The first step involved gaining a better understanding of the artisanal gold mining practices through a review of the scientific literature. This search included databases such as ScienceDirect, PubMed, and Scopus, using keywords such as artisanal gold mining, ASGM, cyanide, cyanide contamination, environmental risk assessment, Burkina Faso, exposure pathways, health risk, and ecological impact.

The second step focused on a detailed understanding of the local context and specific extraction practices. Field visits were conducted at the ASGM sites to carry out on-site observations and to conduct unstructured interviews with the artisanal miners. These visits helped identify major sources of cyanide pollution, including defective cyanidation ponds and uncontrolled discharges into the environment. The review of gold extraction methods—particularly the use of cyanide—allowed for the identification of key exposure pathways among the miners [39]. These findings were then cross-referenced with geographic, demographic, and socio-economic data to identify the at-risk populations.

#### 2.7.2. Design of the Site Conceptual Diagram

The conceptual model was developed to visualize the relationships between cyanide sources, transport mechanisms, exposure pathways, and potential receptors. The site conceptual diagram was based on a framework published by the American Society for Testing and Materials (ASTM) and adapted from [40] to meet the specific requirements of this study. The diagram was modified by adding two columns, “Impacted Environmental Compartments” and “Exposure Mechanisms and Points”, in order to provide additional detail on the exposure processes and affected areas, thereby improving the precision of the exposure analysis.

Using the online software Lucidchart (Lucid Software Inc., South Jordan, UT, USA), cyanide transport flows were mapped from leaching ponds and mine tailings storage areas to human and environmental receptors. Transport mechanisms, such as runoff and infiltration into groundwater, were integrated to illustrate the main dispersion pathways. The developed model identified key exposure routes—including inhalation of hydrogen cyanide gas (HCN), ingestion of contaminated water, and dermal contact with polluted soils and waters—as well as critical receptors, such as mine workers, nearby residents, and vulnerable aquatic ecosystems. This process led to the creation of an initial site conceptual model, which was then submitted for validation.

#### 2.7.3. Validation of the Site Conceptual Model (SCM)

Validation of the conceptual model was carried out through a semi-structured survey with a panel of experts from various disciplines. This approach enabled an assessment of the model’s relevance, robustness, and consistency with field realities and the latest scientific knowledge [11].

**Figure 4:**
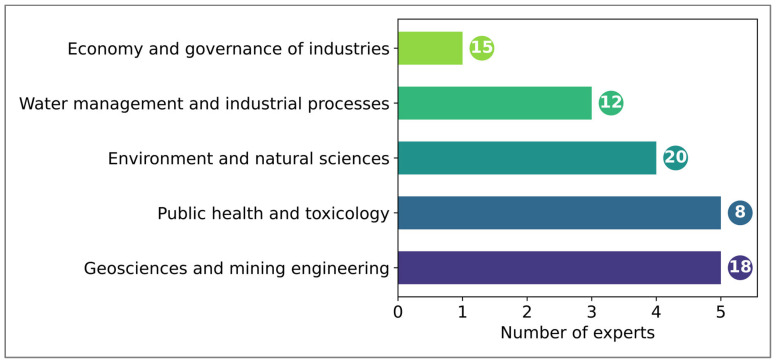
Profiles of the experts surveyed for the validation of the conceptual model (average years of experience represented by bubbles in front of the bars).

A targeted sample was selected to ensure multidisciplinary evaluation. It included experts in artisanal and small-scale gold mining, public health, toxicology, ecotoxicology, environmental engineering, and technical officers from the mining sector (Figure 4). This diversity of expertise ensured a comprehensive review covering technical, health, and ecological dimensions of the model [11].

**Figure 5:**
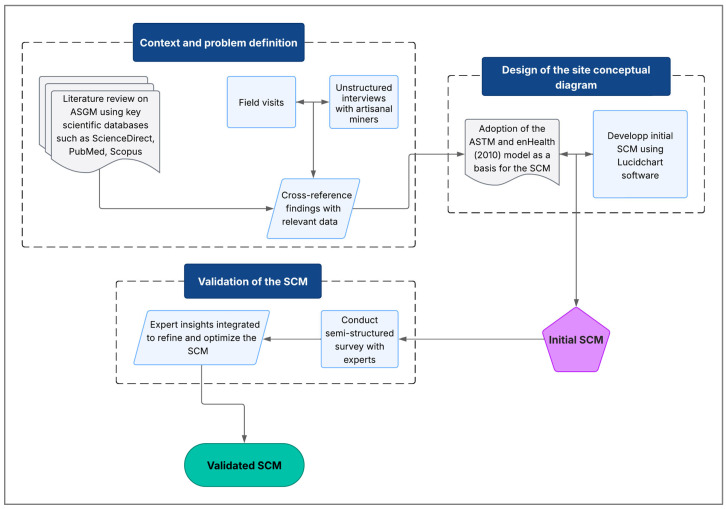
Flowchart illustrating the development, design, and validation process of the site conceptual model (SCM) for assessing cyanide-related exposure risks in ASGM contexts.

Interviews were conducted either in person or remotely, depending on participant availability, and followed a structured guide exploring several aspects of the model. These interviews identified the main sources of pollution, analyzed the dispersion of cyanide in the soil and water, and assessed environmental impacts and the most vulnerable populations. Discussions also addressed the relevance of exposure pathways and the proposed scenarios. Insights from this expert review were integrated to refine the initial conceptual model and to produce an optimized version. Figure 5 provides a schematic overview of the key steps involved in the development, design, and validation of the site conceptual model (SCM) used in this study.

## 3. Results

### 3.1. Quantification of Water Pollution in ASGM Watersheds

**Figure 6:**
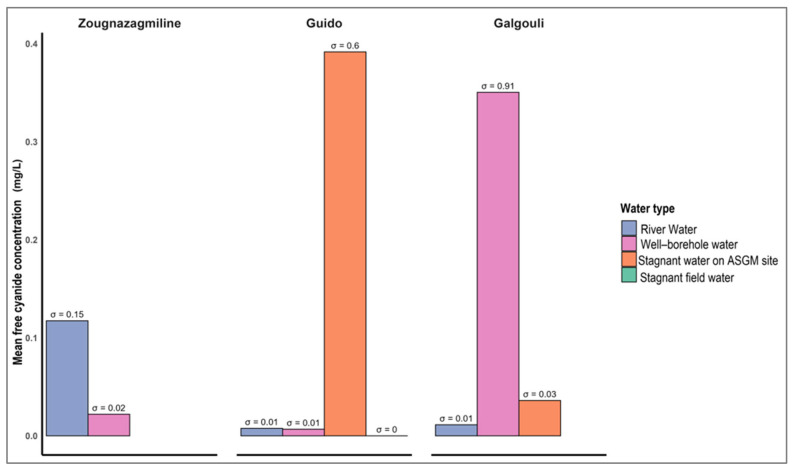
Distribution of free cyanide concentrations in water from the ASGM sites.

Figure 6 shows free cyanide concentrations across the various water matrices from the artisanal mining sites of Zougnazagmiline, Guido, and Galgouli, highlighting the marked variability by site and water type. Overall, 9.26% of the samples exceed the WHO guideline of 0.07 mg/L [41]. At Zougnazagmiline, the values ranged from 0.009 to 0.223 mg/L, with river water showing the highest mean (0.117 mg/L), with lower levels in wells and boreholes (0.022 mg/L). At Guido, concentrations varied between 0 and 1.084 mg/L, peaking in stagnant mining water (0.392 mg/L), while well and river waters remained below 0.01 mg/L. No cyanide was detected in stagnant field waters. Galgouli showed the widest range (0.002–2.42 mg/L), with the highest mean in well–borehole water (0.350 mg/L), moderate levels in stagnant mining and field waters (0.036 and 0.124 mg/L), and the lowest in river water (0.011 mg/L).

An ANOVA was conducted to assess variations in free cyanide concentrations across the different water types at the three sites (Table 1). At Zougnazagmiline, a statistically significant difference was found between water types (F(2,14) = 64.13, *p* < 0.001), with a between-group sum of squares of 0.04 and negligible within-group variance. By contrast, no significant differences were observed at Guido (F(4,10) = 1.24, *p* = 0.35) or Galgouli (F(4,17) = 0.58, *p* = 0.68), despite between-group sums of squares of 0.36 and 0.67, respectively. Within-group variance was higher at these sites (0.72 at Guido and 4.86 at Galgouli).

### 3.2. Quantification of Soil Pollution in the ASGM Watersheds

**Figure 7:**
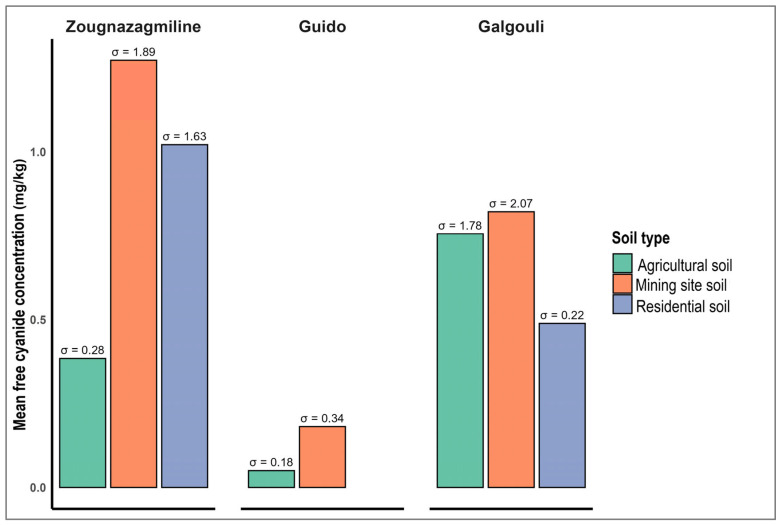
Distribution of free cyanide concentrations in soil from the ASGM sites.

Figure 7 reveals a skewed distribution of free cyanide concentrations in soils from the artisanal gold mining sites in Burkina Faso, with marked variation by soil type and location. Nearly 29.36% of samples exceed the Canadian Council of Ministers of the Environment (CCME) guideline of 0.5 mg/kg for uncontaminated soils [42], indicating localized pollution with potential environmental and health risks. At Zougnazagmiline, concentrations range from 0.04 to 6.25 mg/kg. Mining soils show the highest mean (1.27 mg/kg), followed by residential (1.02 mg/kg) and agricultural soils (0.38 mg/kg), with respective standard deviations of 1.89, 1.63, and 0.28 mg/kg. At Guido, levels are lower (0.00–0.80 mg/kg). The mean free cyanide level in the mining soils is 0.18 mg/kg, slightly above that of the agricultural soils (0.05 mg/kg), with variability reflected in standard deviations of 0.34 and 0.18 mg/kg. At Galgouli, the concentrations range from 0.02 to 9.79 mg/kg. Mining, agricultural, and residential soils show mean values of 0.82, 0.76, and 0.49 mg/kg, respectively, with corresponding dispersions of 2.07, 1.78, and 0.22 mg/kg. Overall, the highest free cyanide levels are consistently observed in the mining soils, confirming their central role in local contamination.

ANOVA results for soil free cyanide concentrations show low statistical variability across soil types within each site (Table 2). At Zougnazagmiline, the F-value is 2.15 with a *p*-value of 0.13; at Guido, F = 1.73 (*p* = 0.20); and at Galgouli, F = 0.14 (*p* = 0.87). In all cases, *p*-values exceed the 0.05 significance threshold, indicating no statistically significant differences in free cyanide concentrations between soil types at the site level.

### 3.3. Land Use Metrics and Cyanide Spatial Distribution in the ASGM Watersheds

The supervised land use and land cover (LULC) classification across the ASGM watershed sub-basins (Galgouli, Guido, and Zougnazagmiline) produced satisfactory results. The analysis focused on four LULC classes, including Agriculture, Built-Up, Natural Land, and Water. Natural land was the dominant land use in Zougnazagmiline (52.78%) and Galgouli (70.63%), whereas agriculture predominated in Guido (68.5%). However, the artisanal gold mining activities were widespread across other land use units, particularly within agricultural fields and often in close proximity to residential areas. The classification performance was evaluated using overall accuracy (OA) and the kappa coefficient (KC), along with producer’s accuracy (PA) and consumer’s accuracy (CA) for each LULC class. Zougnazagmiline and Galgouli showed moderate performance, with lower accuracy in agriculture and water classes, while Guido achieved consistently high scores across all categories. The results are summarized in Table 3.

**Figure 8:**
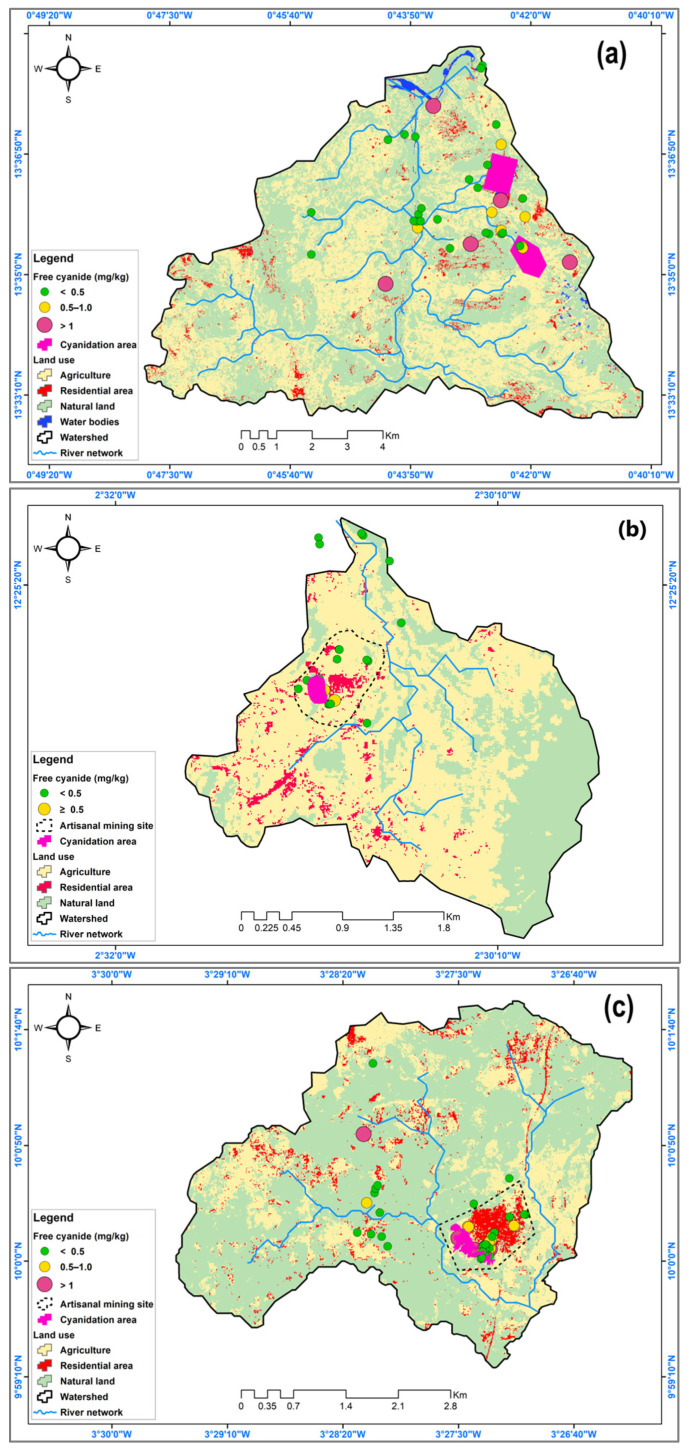
Spatial analysis maps of free cyanide pollution combined with land use in the sites of (**a**) Zougnazagmiline, (**b**) Guido, and (**c**) Galgouli.

A spatial analysis of free cyanide concentrations in the soils from the three study sites—Zougnazagmiline, Guido, and Galgouli—reveals a heterogeneous distribution closely linked to the artisanal mining activities and land use (Figure 8). The mapping results show that contamination is highly concentrated around cyanidation zones, with gradual dispersion into residential and agricultural areas. Although mining zones represent on average only 6% of the watershed areas, they account for the majority of the pollution. At Zougnazagmiline, 46% of the mining soil samples exceed the 0.5 mg/kg threshold, compared to 32% in residential areas and 18% in agricultural zones. At Guido, where mining is less extensive, only 8% of the samples exceed this limit, with 65% of them located within mining areas. In Galgouli, the impact is more widespread, where 27% of agricultural and 39% of residential soils show concerning free cyanide levels, indicating broader pollution dispersion.

### 3.4. Site Conceptual Model

#### 3.4.1. Overview of Cyanide Pathway from the Pollution Source to Affected Entities

Cyanide, widely used at the artisanal gold mining sites in Burkina Faso, is primarily released into the environment from cyanidation ponds. These discharges, carried by rainwater runoff, contaminate various environmental media while directly or indirectly affecting human communities. The artisanal miners, who are in direct contact with cyanide, face immediate exposure risks with potentially severe health consequences.

Contamination then extends to nearby residential areas, where local populations—particularly children—are exposed through contact with polluted water. Agricultural lands are also impacted, as cyanide-laden residue are transported to fields, contaminating food crops and threatening both food security and public health.

Finally, cyanide enters aquatic ecosystems, severely affecting the fauna, especially freshwater fish, which are highly sensitive to this toxic compound. As a result, human populations consuming these fish may also face serious health risks.

#### 3.4.2. Pollution Sources and Transport Mechanisms

Cyanidation ponds, whether active or abandoned, are the primary sources of cyanide pollution at the artisanal and small-scale gold mining (ASGM) sites. These ponds are often rudimentarily constructed without compliance with environmental safety standards, and are unsuitable for the long-term containment of toxic residue. Their poor construction frequently leads to cracks, overflows, or leaks, thereby facilitating cyanide dispersion into the environment [43]. Another major issue lies in the lack of adequate containment measures after abandonment. These ponds are often left untreated and unprotected, prolonging contamination [5].

Secondary pollution sources include uncontrolled mine tailings—also referred to as waste or spoil—often dumped near mining sites or directly on agricultural land. In some cases, these tailings are reused as construction materials [44]. Such practices increase the risk of soil and water pollution, undermining environmental and public health safety in the affected areas [5].

**Figure 9:**
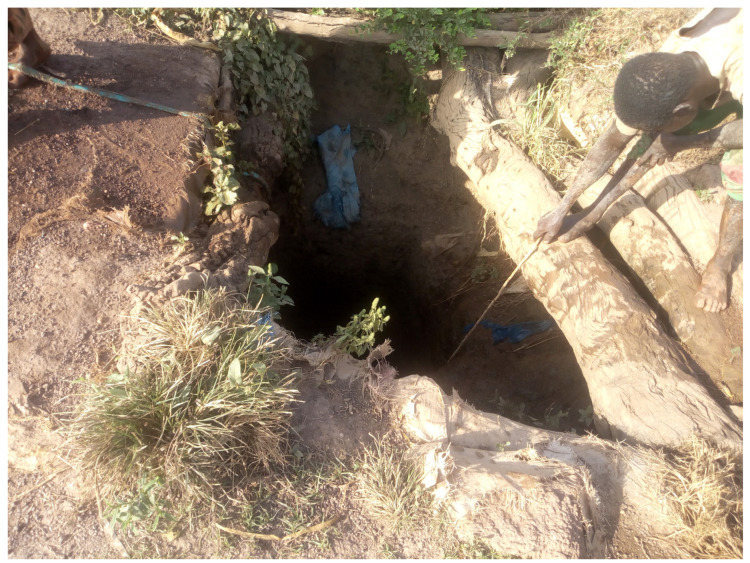
Woman and child collecting water for domestic use from an unprotected well located approximately 400 m from a cyanidation site in the village of Poura (Lat: 11.6240, Long: −2.7657), illustrating potential exposure risks through surface runoff infiltration (November 2021).

Gaseous hydrogen cyanide released during cyanidation can be dispersed by the wind toward nearby communities [9]. However, rainfall runoff remains the principal cyanide transport mechanism. It carries cyanide-rich residue from cyanidation ponds to surface water bodies—such as rivers and reservoirs—and facilitates infiltration into the groundwater [36]. Groundwater is particularly vulnerable, often subjected to dual contamination via infiltration and runoff due to the absence of protective wellheads (Figure 9).

Furthermore, wind can exacerbate contamination by dispersing dry tailings over large areas, a risk heightened in drought-affected regions. The wind also promotes the spread of hydrogen cyanide gas from processing zones to more distant locations [39]. Yet, transport mechanisms are not limited to natural phenomena. Human activities also play a critical role in the spread of cyanide, including such practices as the uncontrolled dumping of tailings on farmland or in watercourses, and the use of contaminated materials for local infrastructure construction, which accelerate cyanide dissemination. These practices may lead to broader and less predictable contamination patterns.

#### 3.4.3. Exposure Pathways and Exposed Populations

At the ASGM sites in Burkina Faso, the first contact between humans and cyanide occurs during its transport and handling. Field observations and previous studies have indicated that miners rarely use protective equipment, primarily due to a lack of awareness about the associated risks. Some workers even handle cyanide with bare hands, without any protection [15]. At this stage, the main exposure routes are dermal contact and accidental ingestion due to poor hygiene practices, such as not washing hands after handling cyanide. The most exposed individuals are those involved in the cyanide transport and the chemical processing of the ore [45].

Two cyanide-based chemical processes are used to recover gold at the ASGM sites in Burkina Faso: the Merrill–Crowe process, which relies on the cementation of gold onto zinc shavings, and the carbon-in-column (CIC) method, which uses activated carbon to adsorb gold–cyanide complexes [4]. The process begins with drying and depositing approximately 4500 to 6000 kg of ore residue into leaching basins [4], followed by the addition of 1 to 1.5 kg of sodium cyanide and 400 to 600 L of water [46]. After 24 h of reaction, the aurocyanide solution is filtered and directed into U-shaped pipes containing the recovery material—zinc or activated carbon—which is then chemically treated to extract the gold [4].

While these processes dissolve gold particles from the ore, they can also release toxic gases, such as hydrogen cyanide (HCN), exposing the miners to inhalation risks. Free cyanide volatilization is more likely in acidic conditions. To prevent this, good mining practices recommend monitoring the pH of the cyanide pulp and adding lime to maintain an alkaline environment. However, the miners at the ASGM sites in Burkina Faso often lack the means to control the pH, and some do not use lime at all. This mismanagement leads to pulp acidification and the release of HCN gas, contributing to atmospheric contamination [3,9,46].

Dermal exposure occurs when handling cyanide pulp or aurocyanide solution, while accidental ingestion may result from inadequate handwashing before meals. Repeated exposures can lead to serious effects, ranging from skin irritation to respiratory and neurological damage.

Communities living near the ASGM sites are mainly exposed through inhalation of the vapors emitted during cyanidation [9]. The dumping of cyanide residue near inhabited areas, and their use as construction materials represent hazardous practices—especially for the children who play on contaminated sand piles and may accidentally ingest the residue [44]. While ingested amounts may be negligible in most children, those with pica—a condition that leads to the consumption of non-food substances—are at greater risk. They are exposed through skin contact and, more critically, through the ingestion of harmful quantities of cyanide-contaminated soil [8,12].

Drinking polluted surface or groundwater is another major exposure route. Dermal contact with these waters during daily activities, such as washing clothes, fishing, or bathing, also poses significant risks [13]. Children, particularly those who swim in contaminated rivers, are especially vulnerable due to their exploratory behaviors, which substantially increase their likelihood of developing serious health effects [47,48].

Beyond these direct pathways, the consumption of plants grown in cyanide-contaminated soil presents an additional risk. Some cyanogenic plants—such as cassava, taro, sorghum, maize, and rice—naturally produce cyanide in the form of cyanogenic glycosides, compounds that can break down and release hydrogen cyanide. Although this natural cyanide is already associated with health risks, studies have shown that such plants can absorb and accumulate additional cyanide from the environment when external concentrations are elevated [49]. Consuming these crops may result in gastrointestinal disorders, severe neurological effects and, in cases of chronic high-dose exposure, even death.

**Figure 10:**
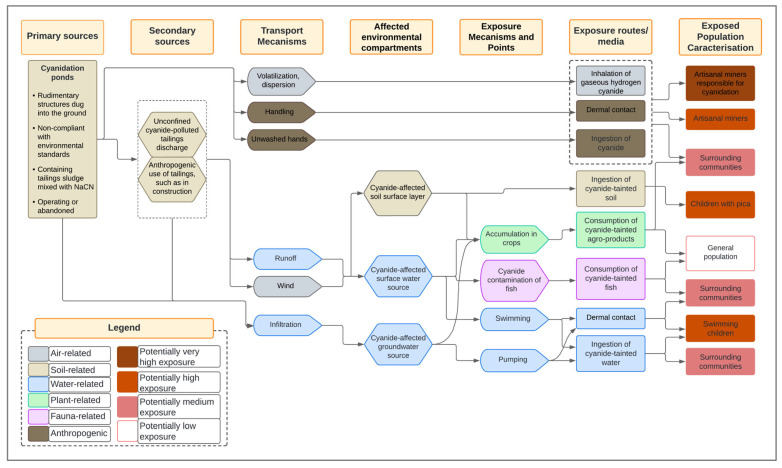
Initial version of the site conceptual model developed for the assessment of human exposure risks to cyanide pollution on the ASGM sites in Burkina Faso.

Additionally, freshwater fish—highly sensitive to cyanide—can accumulate the contaminant in their tissue when exposed to polluted waters. Although cyanide concentrations in fish decline after removal from contaminated environments due to its conversion to less toxic thiocyanate [50], consuming such fish during the contamination period can cause adverse effects in humans, ranging from mild symptoms to severe reactions depending on the ingested dose and individual susceptibility [51]. Figure 10 provides an overview of the initial version of the site conceptual model developed to assess cyanide-related exposure risks in ASGM contexts in Burkina Faso.

#### 3.4.4. Optimization of the Site Conceptual Model Based on Expert Feedback

The analysis of expert feedback revealed converging views on several aspects of the model, as well as criticisms of certain assumptions. Key improvements included the inclusion of storage areas and stockpiles as primary sources of cyanide pollution, along with secondary sources, such as discarded and reused empty containers. The inclusion of tertiary sources was deliberately avoided to maintain conceptual clarity. Experts emphasized the need for greater detail in the representation of cyanide transport mechanisms. Although surface runoff and wind dispersion were already considered, they had not been explicitly illustrated between primary sources and environmental compartments; this was corrected in the revised model. A clearer distinction between transport mechanisms and exposure pathways was also introduced. Atmospheric pollution, previously omitted, was added as an impacted compartment, particularly due to hydrogen cyanide (HCN) volatilization.

**Figure 11:**
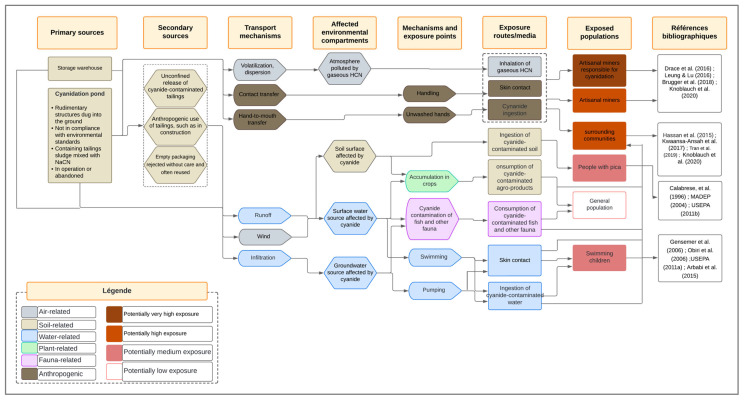
Optimized version of the site conceptual model based on expert feedback for the assessment of human exposure risks to cyanide pollution on the ASGM sites in Burkina Faso [3,8,9,12,13,14,45,46,47,48,52,53,54,55].

While certain experts suggested further specification of exposure routes—such as clarifying ingestion as oral exposure—the model was already explicit about major routes, like ingestion, and additional detail was deemed unnecessary to preserve clarity. Redundant categories of target populations were removed, connector arrows were rearranged to improve readability, and terminology was refined for inclusiveness. For example, “pica children” was replaced with “individuals with pica”, and the heading “Exposed population characterization” was revised to “Exposed populations”. An additional column citing bibliographic references was introduced to justify exposure assumptions. Visual clarity was further enhanced by moving the legend outside the diagram and standardizing arrow formatting. The addition of column borders was rejected to avoid visual clutter. Table 4 summarizes the key recommendations from the expert validation survey, which were integrated to produce the optimized and validated version of the conceptual site model presented in Figure 11. This version provides a robust and operational framework for assessing the health risks related to cyanide pollution from artisanal and small-scale gold mining in Burkina Faso.

## 4. Discussion

### 4.1. Analysis of Cyanide Pollution in Water Resources

Free cyanide concentrations measured in the water samples from the artisanal gold mining sites of Zougnazagmiline, Guido, and Galgouli revealed marked heterogeneity across the sites and the water types (Figure 6). The highest concentrations were recorded in Galgouli, particularly in the waters directly exposed to ore processing activities. This elevated pollution may be explained by the site’s mining intensity and the high gold content of the ore, which likely promotes repeated cyanidation cycles [5]. The groundwater samples from Galgouli reached levels as high as 2.42 mg/L, far exceeding the recommended safety thresholds. This contamination is likely linked to the proximity of boreholes to active processing zones [36], further exacerbated by the shallow depth of the groundwater table [56].

By contrast, cyanide levels measured at Guido and Zougnazagmiline were substantially lower, with average values below 0.5 mg/L across all water types. One-way ANOVA revealed statistically significant differences between the water types only at the Zougnazagmiline site, suggesting that cyanide dispersion is strongly influenced by site-specific hydrological and geographical conditions [36,57,58]. The relative distance between the water sources and the processing areas may also play a role in shaping the cyanide distribution patterns.

The concentrations observed in this study are considerably higher than those previously reported in the ASGM zones of northern Burkina Faso, where free cyanide was undetectable in well water [3]. However, they remain below those reported in the Mbeya mining area in Tanzania, where surface waters contained up to 8 mg/L [59]. Our results are in line with free cyanide levels ranging from 0.004 to 0.45 mg/L recorded in boreholes, wells, dams, and streams in the other studies [54,60].

### 4.2. Soil Cyanide Distribution Across Land Uses in the ASGM Watersheds

Although some samples exceeded the 0.5 mg/kg reference threshold, most soil samples collected across the three sites showed free cyanide concentrations below this limit, indicating localized contamination linked to the ASGM activities (Figure 7). Average concentrations displayed notable variations depending on soil type and location. At Zougnazagmiline, free cyanide levels rose progressively from agricultural soils (0.5 mg/kg) to residential areas (1.0 mg/kg), peaking at 1.25 mg/kg in mining zone soils. In Guido, the values remained low, ranging from 0.06 mg/kg in agricultural soils to 0.18 mg/kg in mining soils. A distinct pattern was observed at Galgouli, where agricultural and mining soils both exhibited relatively high concentrations (0.8 and 0.75 mg/kg, respectively), while residential soils were slightly less contaminated (0.5 mg/kg).

The similarity in free cyanide levels between agricultural and mining soils in some areas likely results from the coexistence of mining and agricultural activities, a common feature of the ASGM zones in Burkina Faso. A statistical analysis of free cyanide concentrations by soil type did not yield significant differences across the sites, with *p*-values of 0.13 for Zougnazagmiline, 0.20 for Guido, and 0.87 for Galgouli—all well above the 0.05 significance threshold. This suggests diffuse contamination, with no statistically meaningful differentiation between land uses.

Measured concentrations in this study were lower than those previously reported in the ASGM zones of northern Burkina Faso, where levels reached up to 20 mg/kg [3]. However, they remain far below the extreme values found in Sekotong, Indonesia, where surface soils contained between 63.93 and 104.08 mg/kg, with even higher concentrations detected at depth [61].

The heterogeneity observed in soil contamination across the three sites appears to result from a combination of factors. These include differences in extraction methods, ore processing techniques, mining intensity, and residue disposal practices [6,36,62]. Moreover, environmental and soil-specific parameters—such as substrate composition, permeability, pH, and local hydrology—likely influence cyanide retention and degradation rates [62,63]. These findings reinforce the need for site-specific assessments when evaluating the environmental risks of artisanal gold mining.

### 4.3. Cross-Cutting Interpretation of Pollution Drivers and Implications

The levels of free cyanide pollution observed at the three study sites appear to reflect not only geographic and hydrological differences, but deeper temporal and economic dynamics shaping ASGM in Burkina Faso. A key factor is the steady and substantial rise in global gold prices over the past two decades. Since April 2001, when gold was valued at approximately USD 260 per ounce, its price has consistently climbed—surpassing USD 1000 in 2008, reaching USD 1900 in 2011, rising to USD 2064 in 2020 during the COVID-19 pandemic, and hitting an all-time high of USD 2400 in April 2024 amid persistent geopolitical tensions [64,65].

This economic trend has fueled the introduction and rapid expansion of cyanidation in Burkina Faso’s ASGM sector since the early 2000s. Cyanide-based processing has made it economically feasible to recover gold from low-grade ores, which were previously discarded and often processed informally by women using traditional processing methods [4,66]. The rising gold prices have also triggered waves of artisanal rushes, resulting in increased mining populations and intensified ore treatment.

Among the three study sites, Galgouli and Zougnazagmiline are the most densely populated, with approximately 2000 miners each, compared to just 500 at the less active Guido site. Galgouli also functions as a strategic hub, linking several neighboring gold mining areas [15,22]. This difference in mining intensity is clearly reflected in environmental outcomes: mean soil free cyanide concentrations at Galgouli, Zougnazagmiline, and Guido were 0.72, 0.71, and 0.09 mg/kg, respectively, with maximum values of 9.79, 6.25, and 0.80 mg/kg in the same order.

These findings point to a strong correlation between increased mining intensity—driven by global market dynamics—and free cyanide pollution accumulation. Nevertheless, the economic benefits of ASGM, particularly in terms of employment and income generation, must be weighed against the environmental and public health risks. Most mining operations are conducted informally and lack sufficient environmental safeguards. Risk mitigation strategies must therefore account for technical feasibility, economic viability, and the social realities of mining-dependent communities.

International comparisons underscore the importance of context-specific approaches. While free cyanide concentrations in Burkina Faso’s groundwater and surface water reached up to 2.42 mg/L, these values remain below those recorded in severely contaminated ASGM areas, such as the Puyango–Tumbes River basin in Ecuador, where concentrations were up to 9088 times higher than the Canadian water quality guideline of 5 µg/L, and 1136 times above the 24-h LC50 threshold for aquatic species (40 µg/L) [67]. Conversely, in the Chinchipe River in Peru, free cyanide levels ranging from 0.034 to 0.04 mg/L remained within the WHO guideline limit for surface water (0.07 mg/L) [68]. These discrepancies reveal the wide variability in cyanide pollution, and reaffirm the necessity of tailoring assessments and interventions to local environmental, economic, and social contexts.

Finally, these variations cannot be attributed solely to differences in mining practices, residue management, or local geological and hydrological characteristics. They also reflect the physicochemical properties of cyanide, a highly unstable compound in the environment. Its volatility, high solubility, and rapid degradation under certain conditions (e.g., pH, light, temperature), as well as its persistence in confined systems, significantly influence its dispersion and retention. The interplay of these factors highlights the critical need for adaptive and site-specific evaluation and mitigation strategies.

### 4.4. Environmental Implications of Land Use Metrics and Cyanide Spatial Patterns in the ASGM Watersheds

Despite the rural and mining-impacted nature of the study area, the classification model performed reliably across all three watersheds (Table 3). The highest accuracy was observed in Guido, where industrial mining infrastructure and sedimentation ponds enhanced the spectral separability of the Built-Up and Water classes. In Zougnazagmiline, the Natural Land and Built-Up areas were well detected, although Agriculture showed lower producer accuracy, possibly due to confusion with the degraded lands near the mining zones [69].

Galgouli, with the lowest classification accuracy, may reflect more heterogeneous land cover and less distinct class boundaries, particularly between Agriculture and Natural Land. This may be due to the widespread presence of mango (*Mangifera indica*) and cashew (*Anacardium occidentale*) orchards, which, although agricultural, share spectral characteristics with natural vegetation [70]. Overall, the integration of NDVI, NDWI, and NDBI improved class differentiation, particularly between natural and human-altered features in these environmentally disturbed settings [71].

The spatial analysis results revealed that the contamination is not confined to mining sites but extends into residential and agricultural areas (Figure 8). The spatial dispersion of free cyanide appears to be governed by several mechanisms, as follows surface runoff, which transports cyanide-laden residue into agricultural lands during rainfall events; infiltration, which promotes deeper soil contamination [72]; and atmospheric dispersion of contaminated dust particles, particularly during the dry season. These processes account for the significant free cyanide concentrations observed in the agricultural and residential soils at Zougnazagmiline and Galgouli, where mining activity is more intense [3].

Contamination of the agricultural soils represents a major environmental and health concern, as it can severely affect crop vitality and compromise the quality of local water resources. The presence of free cyanide in cultivated soils may inhibit plant growth, disrupt nutrient uptake, and reduce agricultural productivity [39,49,73]. In some cases, cyanide may be taken up by the plants and accumulate in the edible tissues, particularly in leafy vegetables and root crops [54]. This potential bioaccumulation could lead to human exposure through the food chain, raising concerns about chronic health effects, especially for vulnerable groups, such as children and pregnant women [54,74].

Similarly, the pollution of residential soils constitutes a direct and continuous threat to human health. Residents may be exposed to cyanide through multiple pathways, including inhalation of contaminated dust, particularly during the dry season, dermal absorption from contact with polluted soil surfaces, especially among children playing outdoors, and incidental ingestion of soil particles or cyanide-contaminated household water. Prolonged exposure through these pathways may contribute to subclinical health effects, such as thyroid dysfunction, impaired cognitive development, and increased risk of chronic diseases [12,13,14,48,55].

The generated maps serve as a strategic tool for policymakers and environmental managers, enabling the identification of priority remediation areas and supporting the optimization of risk management strategies. They also provide a critical scientific basis for the long-term monitoring of cyanide pollution, reinforcing the development of regulatory measures, environmental surveillance systems, and awareness-raising initiatives targeting affected communities.

### 4.5. Site Conceptual Model and Human Exposure Risks

A synthesis of the existing research on cyanide pollution in the artisanal gold mining areas in Burkina Faso reveals a complex chain linking pollution sources to environmental and health impacts. The available studies identify the main cyanide entry points into the environment, particularly poorly constructed and abandoned cyanidation ponds lacking proper containment [43,44]. These structural weaknesses facilitate cyanide dissemination through rainfall runoff, soil and groundwater infiltration, air volatilization, as well as human practices, such as direct discharge and uncontrolled reuse of mining residue [5,9].

Cyanide affects multiple environmental compartments—including agricultural soils, surface and groundwater, and the atmosphere—and also reaches inhabited areas. These overlapping contaminations multiply the human exposure routes, including ingestion of contaminated water, polluted soil, or food from exposed zones, inhalation of toxic vapors, dermal contact, and consumption of agricultural or aquatic products that have bioaccumulated cyanide. The most vulnerable groups include children [8,47], women living near treatment areas, and artisanal mining workers (Figure 10 and Figure 11). This diversity of exposure pathways highlights the need for an integrated risk assessment approach.

However, the literature often addresses these elements in isolation, separating sources, vectors, and effects without offering an overarching perspective [6,75]. This limitation is further compounded by local contextual variability—mining practices, climate conditions, land use—which makes cross-site interpretation difficult. As a result, there is a lack of conceptual tools capable of systematically representing cyanide transfer chains in the Sahelian environments.

In this context, the conceptual site model (CSM) emerges as a structuring tool that connects pollution sources, transport mechanisms, impacted media, exposure pathways, and at-risk groups [35,36]. It supports both integrated risk analysis and the construction of relevant exposure scenarios for quantitative risk assessment [38].

The validation of the CSM by a panel of experts has strengthened its scientific reliability [76,77], confirming the relevance of its core components and suggesting key refinements. The inclusion of secondary sources, such as storage areas and used containers, better reflects field realities, in line with Nikolaidis and Shen [78], who emphasized the importance of accounting for diffuse sources. The incorporation of runoff and wind dispersion—especially relevant in semi-arid climates—enhances the model’s depiction of contaminant transfer dynamics [37,77].

The discussion also addressed the balance between scientific precision and model readability. Although some experts suggested incorporating additional detail regarding exposed groups and exposure pathways, the decision was made to preserve a visually clear and operational tool. This approach reflects the concerns that excessive information can compromise the interpretability and interdisciplinary usability of the conceptual model [37,77].

Finally, the inclusion of a bibliographic reference column reinforces the model’s traceability, consistent with the need to link each connection to a verifiable source [79]. The revised and validated model thus stands as a contextually grounded tool—both structurally useful for exposure scenario analysis and aligned with the local environmental and social context—intended to support both scientific inquiry and operational decision-making.

While the model offers substantial contributions, this study has some limitations. Due to regional insecurity, field access to some previously studied sites was not possible, preventing the collection of updated data. Furthermore, the lack of seasonal or time-series data limited our ability to assess temporal variability in cyanide contamination. Future studies should aim to collect synchronized and seasonally stratified data to enhance temporal comparisons.

## 5. Conclusions

This study provides an integrated assessment of free cyanide contamination and related health risks in three artisanal and small-scale gold mining (ASGM) sites in Burkina Faso. Free cyanide levels exceeded the WHO safety thresholds in 9.26% of the water samples and 29.36% of the soil samples, with maximum concentrations reaching 2.42 mg/L in the groundwater and 9.79 mg/kg in the mining soils. Although the mining zones occupy only about 6% of the watershed areas, they are the primary sources of contamination, which gradually extends into the surrounding residential and agricultural zones, raising public health concerns due to potential chronic exposure to cyanide through the water, soil, and locally grown food.

Beyond the quantification of contamination, a source-to-outcome conceptual model (SCM) was developed and validated through expert consultation to map the cyanide pathways from emission to human exposure. The final model integrates diffuse sources, transport mechanisms, and atmospheric compartments, enhancing its scientific reliability and practical usability.

The SCM serves as a decision-support tool for risk assessment and mitigation planning in ASGM contexts. It is adaptable to other contaminated mining regions, and can help stakeholders prioritize interventions where data and regulatory oversight are limited. Overall, this study highlights the need for targeted, context-specific strategies to reduce cyanide-related risks in the artisanal gold mining communities.

## Figures and Tables

**Table 1:** ANOVA results for free cyanide concentrations in different water types at the ASGM sites.

ASGM Site	Source of Variation	Sum of Squares	df	Mean Square	F	Sig.
Zougnazagmiline	Between water type	0.04	2.00	0.02	64.13	0.00
	Within water type	0.00	14.00	0.00		
Guido	Between water type	0.36	4.00	0.09	1.24	0.35
	Within water type	0.72	10.00	0.07		
Galgouli	Between water type	0.67	4.00	0.17	0.58	0.68
	Within water type	4.86	17.00	0.29		

**Table 2:** ANOVA results for free cyanide concentrations in different soil types at the ASGM sites.

ASGM Site	Source of Variation	Sum of Squares	df	Mean Square	F	Sig.
Zougnazagmiline	Between Soil type	5.67	2.00	2.83	2.15	0.13
	Within Soil type	43.39	33.00	1.31		
Guido	Between Soil type	0.10	1.00	0.10	1.73	0.20
	Within Soil type	1.40	25.00	0.06		
Galgouli	Between Soil type	0.83	2.00	0.42	0.14	0.87
	Within Soil type	128.47	43.00	2.99		

**Table 3:** Classification accuracy and class-wise performance for each watershed.

Watershed	Area (ha)	OA	KC	Class	Land Use (%)	PA	CA
Zougnazagmiline	3501.7	0.77	0.63	Agriculture	45.41	0.60	0.66
				Built-Up	1.21	0.83	0.77
				Natural Land	52.78	0.83	0.80
				Water	0.6	0.73	0.80
Guido	638	0.85	0.74	Agriculture	68.5	0.94	0.83
				Built-Up	1.72	0.77	1.00
				Natural Land	29.78	0.79	0.78
				Water	-	1.00	1.00
Galgouli	1614	0.74	0.60	Agriculture	26.39	0.64	0.78
				Built-Up	2.97	0.85	0.78
				Natural Land	70.63	0.73	0.66
				Water	-	0.75	0.60

**Table 4:** Synthesis of expert feedback and corresponding adjustments to the site conceptual model.

Model Section	Expert Feedback	Action Taken
Pollution Sources	Storage areas and warehouses were not included in the model.	Integration of storage areas and warehouses as pollution sources.
	The absence of secondary sources, such as discarded and often reused empty containers, needs to be addressed.	Addition of empty containers as secondary sources of pollution.
	The inclusion of tertiary pollution sources was suggested.	Proposal rejected to avoid overloading the model.
Transport Mechanisms	Cyanide transport and transfer mechanisms need further detail.	Current level of detail maintained to avoid overcomplicating the model.
	Runoff and wind dispersion mechanisms from primary sources to environmental compartments were not considered.	Addition of runoff and wind transport to the model.
	Some transport mechanisms are confused with vectors, which is misleading.	Clarification of the distinction between transport mechanisms and vectors.
Impacted Environmental Compartments	Atmospheric pollution was not considered an affected compartment.	Addition of the atmosphere as an impacted environmental compartment.
Exposure Mechanisms and Points	Exposure routes should be more clearly defined, e.g., specifying that ingestion refers to the digestive route.	Current wording maintained to preserve model conciseness.
Exposure Pathways/Media	Certain target populations, such as nearby communities, appear multiple times, causing unnecessary repetition.	Reorganization of the diagram to eliminate redundancy and optimization of arrow placements.
Exposed Populations	The title “Characterization of the exposed population” is inappropriate.	Replaced with “Exposed Populations” for greater clarity.
	The use of color to classify exposure levels is not scientifically valid without supporting references.	Addition of a column for bibliographic references to justify exposure levels.
	The term “Pica children” should be replaced with “Individuals with pica”, as the condition can also affect adults, particularly pregnant women.	Terminology revised to better reflect the diversity of affected individuals.
	The term “Artisanal miners” may cause confusion and should be clarified.	Replaced with “Mining workers” or “Artisanal mining workers” to avoid ambiguity.
Model Formatting	Arrows should be standardized to prevent visual confusion.	Harmonization of arrows for improved readability.
	The legend is poorly positioned and may appear to be part of the diagram.	Relocated the legend outside the diagram for better clarity.
	Adding borders to the diagram columns was suggested to better structure the model.	Suggestion rejected to avoid cluttering the layout due to border overlaps with arrows.

## Data Availability

The datasets generated and/or analyzed during the current study are available from the corresponding author upon reasonable request.
